# Copeptin for risk stratification in non-traumatic headache in the emergency setting: a prospective multicenter observational cohort study

**DOI:** 10.1186/s10194-017-0733-2

**Published:** 2017-02-13

**Authors:** Claudine Angela Blum, Bettina Winzeler, Nicole Nigro, Philipp Schuetz, Silke Biethahn, Timo Kahles, Cornelia Mueller, Katharina Timper, Katharina Haaf, Janina Tepperberg, Margareth Amort, Andreas Huber, Roland Bingisser, Peter Stephan Sándor, Krassen Nedeltchev, Beat Müller, Mira Katan, Mirjam Christ-Crain

**Affiliations:** 1 Division of Endocrinology, Department of Internal Medicine, University Hospital Basel, Basel, Switzerland; 2 Department of Neurology, Medical University Clinic, Kantonsspital Aarau, Aarau, Switzerland; 3 Internal Medicine, Medical University Clinic, Kantonsspital Aarau, Tellstrasse, CH-5001 Aarau, Switzerland; 4 Clinic of Neurology, University Hospital Basel, Basel, Switzerland; 5 Center of Laboratory Medicine, Kantonsspital Aarau, Aarau, Switzerland; 6 Emergency Department, University Hospital Basel, Basel, Switzerland; 7 Neurology, RehaClinic AG, Bad Zurzach, Switzerland; 8 Clinic of Neurology, University Hospital Zürich, Zürich, Switzerland; 9 Max-Planck-Institute for Metabolism Research, Cologne, Germany

**Keywords:** Headache, Head pain, Emergency, Biomarker, Copeptin, Vasopressin

## Abstract

**Background:**

In the emergency setting, non-traumatic headache is a benign symptom in 80% of cases, but serious underlying conditions need to be ruled out.

Copeptin improves risk stratification in several acute diseases. Herein, we investigated the value of copeptin to discriminate between serious secondary headache and benign headache forms in the emergency setting.

**Methods:**

Patients presenting with acute non-traumatic headache were prospectively enrolled into an observational cohort study. Copeptin was measured upon presentation to the emergency department. Primary endpoint was serious secondary headache defined by a neurologic cause requiring immediate treatment of the underlying disease. Secondary endpoint was the combination of mortality and hospitalization within 3 months. Two board-certified neurologist blinded to copeptin levels verified the endpoints after a structured 3-month-telephone interview.

**Results:**

Of the 391 patients included, 75 (19%) had a serious secondary headache. Copeptin was associated with serious secondary headache (OR 2.03, 95%CI 1.52–2.70, *p* < 0.0001). Area under the curve (AUC) for copeptin to identify the primary endpoint was 0.70 (0.63–0.76). After adjusting for age > 50, focal-neurological abnormalities, and thunderclap onset of symptoms, copeptin remained an independent predictive factor for serious secondary headache (OR 1.74, 95%CI 1.26–2.39, *p* = 0.001). Moreover, copeptin improved the AUC of the multivariate logistic clinical model (p-LR-test < 0.001).

Even though copeptin values were higher in patients reaching the secondary endpoint, this association was not significant in multivariate logistic regression.

**Conclusions:**

Copeptin was independently associated with serious secondary headache as compared to benign headaches forms. Copeptin may be a promising novel blood biomarker that should be further validated to rule out serious secondary headache in the emergency department.

**Trial registration:**

Study Registration on 08/02/2010 as NCT01174901 at clinicaltrials.gov.

**Electronic supplementary material:**

The online version of this article (doi:10.1186/s10194-017-0733-2) contains supplementary material, which is available to authorized users.

## Background

Headache has a high prevalence in the general population and presents an important public health problem [1].

Non-traumatic headache has a prevalence of 0.5 to 4.5% in patients presenting to the emergency department (ED), and it remains a major diagnostic challenge [2–5]. The majority of these patients have benign headache. However, in 3.8 – 20% of headache patients, a headache secondary to a potentially life-threatening condition is diagnosed with immediate implications for therapeutic interventions. These include subarachnoidal hemorrhage, cerebral aneurysm, intracranial bleeding, sinus vein thrombosis, temporal arteriitis, or meningitis [2, 5–7]. It has been shown by various authors that red flags like age >50 years, focal-neurological signs, thunderclap onset of symptoms or altered mental status have a remarkable negative predictive value around 0.98–0.99, however with varying sensitivities (between 0.39 and 100%) and specificities (between 65 and 98%) [7–11]. To improve the differentiation of benign headaches from secondary, potentially serious headache forms, various clinical algorithms which include these red flags have been proposed, but they are only inconsistently applied in clinical practice [7, 12]. Therefore, the use of biomarkers has potential merits as diagnostic tools.

Only few biomarkers have been investigated in headache, [13–22] and except D-dimer in specific vascular etiologies, [14, 17] they have not found their way into emergency algorithms. To our knowledge, there is no data on biomarkers for discriminating between serious secondary and benign headache forms.

Copeptin is a hypothalamic stress hormone that mirrors the individual stress level even more subtly than circulating cortisol [23]. In its function as a stress hormone, copeptin serves as a prognostic marker in various acute disease states, such as cerebrovascular event, myocardial infarction or pneumonia [24]. Apparently, copeptin allows tapping an endogenous information system of our body that, through mechanisms still poorly understood, assesses the severity of damage [25].

We herein prospectively evaluated the use of copeptin for discriminating between benign headache forms and serious secondary headache, and the degree of association with serious secondary headache.

## Methods

### Study design and participants

This multicenter observational study was performed at the ED of two tertiary care hospitals in northern Switzerland from October 2010 to March 2013.

All patients with current non-traumatic headache as a chief complaint presenting to the ED or as an emergency to the medical or neurological walk-in clinic of the two tertiary centers were eligible and screened.

Exclusion criteria were trauma within 7 days, chronic headache defined as > 3 months duration, age < 18 years, missing informed consent, and inability to comply with study procedures due to insufficient knowledge of the German language.

Chronic headache with an acute exacerbation was not an exclusion criterion.

### Clinical variables

Recruitment was during 24 h and on weekdays and weekends.

After informed consent was given by the patient, baseline data were assessed by questionnaire at the time of the acute presentation. This included medical history, age, gender, body mass index, ethnicity and country of origin, past medical history, smoking history, current medication, alcohol and coffee consumption (units per day), and substance abuse. Clinical headache presentation and headache history was assessed by detailed standardized, validated questionnaire containing the International Classification of Headache Disorders (ICHD) [26] and included depression screening by Primary Care Evaluation of Mental Disorders (PRIME-MD) questionnaire [27] and categorization by a validated, diagnostic algorithm into one of four clinical scenarios (Thunderclap headache, meningitis, new or worsening headache, headache similar to previous episodes) as described by Grimaldi et al. [7]. Disease severity, quality of daily living, and interference of symptoms with quality of life was assessed using the standardized, validated migraine disability assessment (MIDAS) [28].

Clinical items assessed by the treating physician were recorded at the time of the acute presentation and included physical examination, neurological assessment, Glasgow Coma Scale, blood pressure, pulse rate, and body temperature.

### Blood sampling and assays

Blood samples for copeptin measurement were drawn on admission in the emergency department and were either obtained from an indwelling venous catheter or by venous puncture. Blood samples were immediately centrifuged and stored in the refrigerator. Apart from blood sampling for study purposes, routine blood sampling was performed at the discretion of the treating physicians and was performed in 354 (91%) patients.

At ED discharge, the diagnostic work-up was recorded, including neuroimaging, lumbar puncture and additional disease-specific evaluations in secondary headaches.

Blood Plasma was frozen at −70 °C. Copeptin levels were measured by batch analysis with a commercial chemiluminescence sandwich immunoassay (B.R.A.H.M.S LUMItest CT-proAVP, B.R.A.H.M.S AG, Hennigsdorf/Berlin, Germany), as described in detail elsewhere, with a lower detection limit of the assay of 0.4 pmol/L and the functional assay sensitivity (<20% interassay CV) of < 1 pmol/L) [29, 30]. The treating physicians and the study team were blinded to copeptin levels at all times.

### Follow-up

At 3 months, a structured follow-up telephone interview was performed to assess course and outcome of the disease. The earliest possible contact was 14 days before this time point. If the patient was not reached until 4 weeks after the scheduled interview, his primary care physician was contacted for follow-up information. If the patient had consulted the hospital since study inclusion and no other data was available, this date was taken as time of last follow-up. To minimize loss of follow-up, the study team tried to re-contact the patient until the last scheduled follow-up interview in June 2013. The final headache diagnosis and the judgement whether the headache had been serious or not was made in each patient after the follow-up interview by two independent physicians by chart review and verified by a board-certified neurologist. Disagreements between study physicians were resolved by a final adjudication of an independent neurologist. They were all blinded to copeptin levels.

### Outcomes

The objective of this study was to evaluate copeptin as a marker for risk stratification in non-traumatic headache, namely for ruling out serious secondary causes of headache. The primary endpoint was serious secondary headache as opposed to benign headache.

Our hypothesis was that based on previous studies on copeptin in other diseases, [29, 31–34] a cutoff of ≤ 5 pmol/l would have a sensitivity of ≥ 97% for ruling out serious secondary headache causes, and that a cutoff of ≥ 20 pmol/l would have a specificity of ≥90% for the presence of serious secondary NTH.

The secondary endpoint was the combined endpoint of death or hospitalization of any cause within 3 months.

Headache was defined according to the International Headache Society as pain located above the orbitomeatal line. A serious secondary headache was defined as a headache with a neurologic cause as listed in the ICHD-II criteria [9] requiring subsequent treatment of the underlying disease or condition, which, if left untreated, would have the potential risk of permanent damage or death(see also Appendix). All other patients with an uneventful 3-month follow-up were diagnosed with benign headache.

Our gold standard for the final diagnosis of headache was the clinical diagnosis according to ICHD-II criteria after 3 months, verified by a board-certified neurologist. Headaches which clearly belonged to the primary headache entities but did not fulfill the full diagnostic criteria were classified as “primary headache, not classified”, e.g. a first episode of migraine, which did not yet fulfill the criterion of at least 3 episodes.

As this study was started before the current version ICHD3-beta was in place, we continued to use the ICHD-II classification for consistency throughout the whole study.

### Analysis

Discrete variables are summarized as counts (percentage), and continuous variables as medians and interquartile ranges(IQRs). Frequency comparison was done by chi-square test. Two-group comparisons of continuous variables were performed using Student’s *t*-test or the Mann–Whitney-U test, depending on their distribution. Receiver operating characteristic (ROC) curve for copeptin was calculated, and the area under the ROC curve (AUC) was used to assess the discriminatory potential of copeptin to accurately identify patients with serious secondary headache [35]. Further, we calculated logistic regression models to estimate the magnitude of the association between copeptin and the pre-specified endpoints. To assess the independence of this association from other risk scores and important outcome predictors (chosen from the published literature) we calculated multivariable regression models and added all parameters based on their association in the univariate models (p-value cut off for further evaluation was <0.05). This included age > 50 years, sudden onset of headache, an abnormal neurological examination, [11] clinical scenario 4 (history of previous similar headache) for the clinical model (multivariate model 1), [7] D-dimer, fibrinogen, leukocytes and C-reactive protein [13, 36] for the laboratory model (multivariate model 2). To avoid overfitting, we calculated two final models, thus no more than one variable per ten events were included in these final two models. In addition, we compared the ROC curves based on the logistic models with and without copeptin using the likelihood ratio test [37]. All statistical tests were 2-tailed. *p* < 0.05 was considered significant. STATA 12.1 (StataCorp LP, College Station, TX, USA) was used for data analysis.

### Sample size considerations

Based on the patient numbers from previous years, about 400 patients per year were estimated to be eligible for this study. With an estimated informed consent rate of 90–95% and an estimated loss of follow-up of 5–10%, 360 patients were estimated to complete the study. According to pre-existing literature, 10–20% of these 360 patients would present with serious non-traumatic headache, thus 50–60 patients would reach the primary endpoint. Based on the literature and pilot data [25], a mean copeptin difference of 15 pmol/L (SD of 30 pmol/L) between these groups was expected. This difference would be detected with a power of 80%.

## Results

### Characteristics of study subjects

**Fig. 1:**
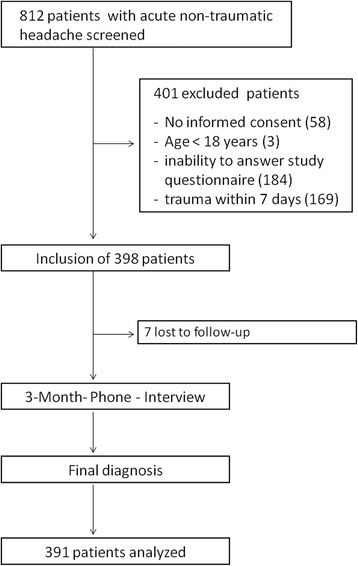
STROBE study flow diagram

From October 2010 to March 2013, 398 patients were enrolled into the CoHead-Study (see Fig. 1 for study flow chart). The 3-month telephone interviews were terminated in July 2013. 7 patients were lost to follow-up and were therefore not included in the analysis.

Baseline characteristics are shown in Table 1. Median age was 41 (IQR 30–57), 146 patients (37%) were men. Median headache duration was 3 days (IQR 0.5–7); median pain strength on Visual Analogue Scale (VAS) was 8 (IQR 6–9). At discharge, a primary headache form was diagnosed in 219 (56%) patients, and secondary headache in 172 (44%) patients, of which 75 (19.2%) were classified as serious.

**Table 1:** Baseline characteristics^a^

Baseline characteristics	All patients (*n* = 391)	Benign headache (*n* = 316)	Serious secondary headache (*n* = 75)
Age (years)	41 (30 – 57)	39 (28 – 54)	56 (40 – 66)
Male sex	146 (37.3%)	107 (33.9%)	39 (52.0%)
Comorbidities			
Hypertension	51 (13.0%)	36 (11.4%)	15 (20.0%)
Coronary heart disease	17 (4.3%)	8 (2.5%)	9 (12.0%)
Atrial fibrillation	2 (0.5%)	1 (0.3%)	1 (1.3%)
Cerebrovascular ischemic disease	2 (0.5%)	0	2 (2.7%)
Chronic obstructive lung disease	1 (0.3%)	1 (0.3%)	0
Multiple sclerosis	2 (0.5%)	2 (0.6%)	0
Chronic pain syndrome	7 (1.8%)	6 (1.9%)	1 (1.3%)
Depression	17 (4.3%)	16 (5.1%)	1 (1.3%)
Epilepsy	9 (2.3%)	3 (0.9%)	6 (8.0%)
Associated headache symptoms			
Nausea	214 (54.7%)	178 (56.3%)	36 (48.0%)
Vomiting	106 (27.1%)	83 (26.3%)	23 (30.7%)
Visual aura	124 (31.7%)	107 (33.9%)	17 (22.7%)
Photophobia	190 (48.6%)	160 (50.6%)	30 (40.0%)
Thunderclap onset headache	67 (17.1%)	39 (12.3%)	28 (37.3%)
Nuchal rigidity	10 (2.6%)	6 (1.9%)	4 (5.3%)
Scenario^b^			
1: Worst headache ever	67 (17.1%)	39 (12.3%)	28 (37.3%)
2: fever/neck stiffness	26 (6.6%)	21 (6.6%)	5 (6.7%)
3: recent worsening	169 (43.2%)	141 (44.6%)	28 (37.3%)
4: history of previous similar headache	112 (28.6%)	99 (31.3%)	13 (17.3%)
5: none of the above	17 (4.3%)	16 (5.1%)	1 (1.3%)
Clinical findings			
Systolic blood pressure (mmHg)	134 (121 – 148)	131 (120 – 145)	139 (125 – 159)
Diastolic blood pressure (mmHg)	80 (71–88)	80 (70 – 87)	78 (73 – 89)
Temperature (°C)	37 (36.7–37.4)	37.0 (36.7–37.4)	37.1 (36.7–37.4)
Headache duration (days)	3 (0.5–7)	2 (1 – 7)	3 (1 – 6)
Visual Analogue Scale (0–10) for pain at ED	8 (6 – 9)	8 (6 – 9)	8 (5 – 10)
Focal-neurological finding	74 (18.9%)	46 (14.6%)	28 (37.3%)
Immediate hospitalization	61 (15.6%)	39 (12.3%)	22 (29.3%)

^a^data are shown as median (IQR) or n (%)

^b^clinical scenarios according to Grimaldi et al. [7]

Scenario 1 (worst headache ever): thunderclap-onset headache suggestive for subarachnoidal bleeding

Scenario 2 (fever/neck stiffness): potential meningitis

Scenario 3 (recent worsening) corresponds to tumor or temporal arteriitis

Scenario 4 (history of previous similar headache) corresponds to a primary headache form

The group of serious secondary headache was heterogeneous: 8 patients (2%) had subarachnoidal hemorrhage, 7 (1.8%) sinus vein thrombosis, 10 (2.6%) intracranial bleeding, and 7 (1.8%) had viral meningitis (detailed diagnoses are shown in Table 2).

**Table 2:** Headache classification

Headache classification (*n* = 391)	*n* (%)
Primary headache	219 (56.0)
- Primary headache, not classified	38 (9.7)
- Migraine	114 (29.1)
- Tension-type headache	51 (13.0)
- Cluster headache	4 (1.0)
- Trigeminus neuralgia	6 (1.5)
- Medication overuse headache	6 (1.5)
Secondary headache	172 (44.0)
- Not serious^a^	97 (24.8)
- Serious:	75 (19.2)
- Subarachnoidal bleeding	8 (2.0)
- Sinus vein thrombosis	7 (1.8)
- Intracerebral bleeding	10 (2.6)
- Cerebral tumor	6 (1.5)
- Cerebral ischemia	6 (1.5)
- Dissection of carotid artery	4 (1.0)
- Temporal arteriitis	3 (0.8)
- Bacterial meningitis	1 (0.3)
- Viral meningoencephalitis	7 (1.8)
- Neuroinflammatory disease^b^	4 (1.0)
- Chronic subdural hematoma	5 (1.3)
- High intracranial pressure	3 (0.8)
- Low intracranial pressure	3 (0.8)
- Hypertensive urgency	1 (0.3)
- Associated with epileptic seizure	5 (1.3)
- Herpes zoster with cranial nerve affection	1 (0.3)
- Acute glaucoma	1 (0.3)

^a^31 systemic infections, 2 electrolyte disorders, 23 headaches due to teeth, facial skin or otorhinolaryngologic aetiology, 6 cases of arterial hypertension, 5 muscular headaches, 4 psychosomatic causes, 1 medication side effect, 1 multifactorial headache. 4 residual post-traumatic headaches, 8 hypoliquorrhea syndromes where no measures were necessary, 1 residual postoperative headache, 7 mild viral meningitis without detection of any pathogen, 1 pseudotumor cerebri without necessity of intervention, 1 patient each with headache secondary to Bell’s palsy, Tolosa Hunt syndrome, and polyneuritis cranialis

^b^Neuritis vestibularis, Miller-Fisher syndrome, clinically isolated syndrome, and retrobulbar neuritis

Median time to follow-up was 99 days (IQR 91–118; range 9–632). Two patients (0.5%) died during follow-up. Both had initially presented with serious secondary headache: one with subarachnoidal bleeding and one with bacterial meningitis. Hospitalization rate was 94 patients (24.0%), of which 61 (15.6%) were hospitalized immediately following study inclusion.

## Main results

### Association of copeptin with serious secondary headache

**Fig. 2:**
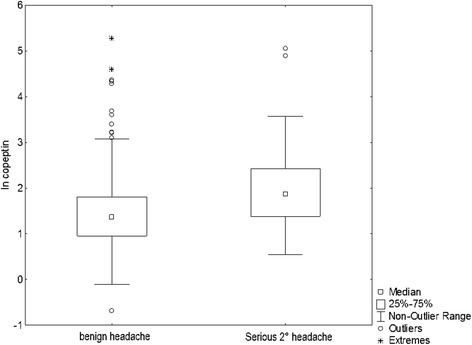
Boxplot of copeptin values

Copeptin levels were higher in serious secondary headache forms as compared to all other headache forms (median 6.44 pmol/L (IQR 3.97–11.29) vs. 3.89 pmol/L (IQR 2.59–6.10)(*p* < 0.0001), see Fig. 2). The AUC for copeptin to detect serious secondary headache was 0.70 (95% confidence interval (CI) 0.63–0.76). At the predefined rule-out cutoff for serious secondary headache of 5.0 pmol/L, copeptin had a sensitivity of 64.4%; at the predefined rule-in cutoff of 20 pmol/L, copeptin had a specificity of 95.3% to rule in a serious secondary headache. At the cutoff of 2.5 pmol/L, copeptin had a sensitivity of 91.8% to rule out serious secondary headache, i.e. a negative predictive value of 0.93. For different copeptin cutoffs, see Table 3.

**Table 3:** Sensitivity and Specificity of copeptin to predict serious secondary headache at different cutoffs

Copeptin value	Sensitivity (%)	Specificity (%)
2.5 pmol/l	91.8	23.3
5.0 pmol/l	64.4	66.7
10.0 pmol/l	27.4	89.9
20.0 pmol/l	12.3	95.3
30.0 pmol/l	5.5	96.5

In univariate analysis, copeptin was associated with serious secondary headache with an OR of 2.03 (95%CI 1.52–2.70, *p* < 0.0001). Other strong predictors were age >50 years (OR 2.83, 95%CI 1.69–4.74, *p* < 0.0001), an abnormal neurological exam (OR 3.50, 95%CI 1.99–6.14, *p* < 0.0001), and thunderclap onset of symptoms (OR 4.23, 95%CI 2.38–7.52, *p* < 0.0001; for detailed results of the univariate analysis, see Table 4a).

**Table 4:** Main results: univariate and multivariate analysis of primary and secondary endpoint*

a. Univariate analysis of primary and secondary endpoint						
	Serious secondary headache			Composite endpoint mortality or hospitalization		
Predictors	OR	95%CI	*p*	OR	95%CI	*p*
Copeptin (ln^a^)	2.03	1.52–2.70	<0.0001	1.45	1.12.1.88	0.005
Age > 50	2.83	1.69–4.74	<0.0001	2.77	1.72–4.46	<0.0001
Male gender	2.12	1.27–3.52	0.004	1.41	0.88–2.27	0.150
Focal-neurological symptoms	3.50	1.99–6.14	<0.0001	4.23	2.47–7.24	<0.0001
Thunderclap onset	4.23	2.38–7.52	<0.0001	3.04	1.75–5.29	<0.0001
Scenario 4*	0.46	0.24–0.87	0.018	0.56	0.32–0.97	0.040
D-Dimer (ln^a^)	1.50	1.00–2.27	0.052	1.28	0.87–1.89	0.205
Fibrinogen (ln^a^)	2.95	1.10–7.89	0.031	2.29	0.91–5.80	0.080
C-reactive protein (ln^a^)	1.06	0.87–1.30	0.544	1.31	1.07–1.60	0.010
Leukocyte count (ln^a^)	2.04	1.01–4.13	0.046	0.63	0.34–1.16	0.136
b. Multivariate model 1: Copeptin and clinical variables						
	Serious secondary headache			Composite endpoint mortality or hospitalization		
Predictors	OR	95%CI	Predictors	OR	95%CI	*p*
Copeptin (ln^a^)	1.73	1.26–2.40	0.001	1.23	0.91–1.65	0.173
Age > 50	2.25	1.28–3.96	0.005	2.34	1.41–3.89	0.001
Male gender	1.84	1.04–3.26	0.035			
Focal-neurological symptoms	2.63	1.40–4.92	0.003	3.41	1.94–6.02	<0.0001
Thunderclap onset	2.90	1.49–5.65	0.002	2.21	1.18–4.15	0.013
Scenario 4^b^	0.73	0.35–1.50	0.388	0.81	0.43–1.51	0.503
c. Multivariate model 2: Copeptin and laboratory variables^a^						
	Serious secondary headache**			Composite endpoint mortality or hospitalization***		
Predictors	OR	95%CI	*p*	OR	95%CI	*p*
Copeptin (ln)	1.80	1.33–2.44	<0.0001	1.39	1.0–1.94	0.050
Fibrinogen (ln)	1.22	0.93–1.61	0.158			
Leukocyte count (ln)	1.78	0.82–3.83	0.143			
C-reactive protein (ln)				1.27	1.04–1.56	0.021

*n = 75 events for the primary endpoint, *n* = 94 events for the secondary endpoint

^a^All laboratory values were transformed by natural logarithm (ln)

^b^Scenario 4 of Grimaldi et al. [7]: previous headache history presenting with a similar episode

For model 2, we performed a subgroup analysis of **n = 327 and ***n = 279 patients with available values of fibrinogen, leukocyte count, and C-reactive protein

We included the strongest clinical risk factors from the univariate analysis along with copeptin in a multivariate model. After adjustment, copeptin remained independently associated with serious secondary headache (OR 1.74, 95%CI 1.26–2.39, *p* = 0.001). The clinical factors age > 50 years (OR 2.25, 95%CI 1.28–3.96), male gender (OR 1.84, 95%CI 1.27–3.52), focal-neurological findings (OR 2.63, 95%CI 1.99–6.14), and thunderclap onset (OR 2.89, 95%CI 2.38–7.52) also remained associated with serious secondary headache (for detailed values, see Table 4b).

Moreover, copeptin improved the AUC of the multivariate logistic clinical model (model without copeptin: AUC 0.735 (95%CI 0.664–0.807), model with copeptin: AUC 0.7544 (95% CI0.683–0.825), p-LR-test < 0.001).

In a second multivariate model, laboratory values including fibrinogen, leukocytes and copeptin were assessed. Only copeptin remained associated with serious secondary headache (OR 1.80, 95%CI 1.33–2.44, *p* < 0.0001; for detailed values, see Table 4c).

### Association of copeptin with mortality or hospitalization

In the 94 (24.0%) patients reaching the composite endpoint death or hospitalization of any cause within 3 months, copeptin values were higher as compared to patients without these events (median 6.44 pmol/L(IQR 3.97–11.29) vs. 3.89 (IQR 2.59–6.01; *p* = 0.0006). The AUC for the composite endpoint was 0.62 (95%CI 0.56-0.68). In univariate logistic regression analysis, copeptin was a predictor for the composite endpoint (OR 1.45, 95%CI 1.12–1.88, *p* = 0.005).

Other strong predictors were age > 50 years (OR 2.77, 95%CI 1.72–4.46), an abnormal neurologic exam (OR 4.23, 95%CI 2.47–7.24), thunderclap onset of headache (OR 3.04, 95%CI 1.75–5.29), clinical scenario 4 (history of previous similar headache; OR 0.56, 95%CI 0.32–0.97), and C-reactive protein (OR 1.31, 95%CI 1.07–1.60; see also Table 4a). After inclusion of the significant clinical predictors from the univariate analysis along with copeptin in a multivariate model, only age >50 (OR 2.34, 95% CI 1.41–3.89), focal-neurological findings (OR 3.41, 95% CI 1.94–6.02) and thunderclap onset (OR 2.21, 95% CI 1.18–4.15) remained associated with the composite endpoint death or hospitalization, but not copeptin (for detailed values, see Table 4b). In the second multivariate model, C-reactive protein (OR 1.27, 95%CI 1.04–1.56) but not copeptin (OR 1.39 (95%CI 1.0–1.94), *p* = 0.05), remained associated with death or hospitalization, (For detailed values, see Table 4c).

### Limitations

We are aware of potential limitations.

First, we included patients with headache irrespective of the underlying cause or disease. This leads to an inhomogeneous case mix with small numbers of each underlying cause, both in benign and in serious secondary headaches. However, using broad inclusion criteria reduces selection bias and, arguably, represents the true clinical population admitted to an emergency setting with acute headache.

Second, our gold standard for the final diagnosis of headache was the clinical diagnosis according to ICHD criteria after 3 months, verified by a board-certified neurologist. We abstained from brain imaging due to organizational and monetary restraints. In patients without brain imaging, there remains a small risk that a cerebral pathology may have been missed. However, we considered it to be rather unlikely that a clinically relevant cerebral pathology would remain unnoticed at 3 months. Moreover this would have introduced a bias towards the null hypothesis thus underestimating the real association.

Third, even though we aimed to recruit 24 h per day and 7 days per week, sampling error is possible, as patients arriving at night might have been missed by the staff on duty. Furthermore, this cohort represents an emergency department population and not the general headache patient.

Fourth, as copeptin rises after a stress stimulus independent from its cause, potential confounding factors have to be ruled out, such as hypoxia or hypovolemia, and inflammatory states [38–40].

## Discussion

Our main finding is that copeptin was higher in serious secondary headaches requiring subsequent treatment of the underlying disease, as compared to benign headache forms. Importantly, after adjusting for the classical clinical risk factors age >50, sudden onset of headache and abnormal neurological exam [11], copeptin remained an independent risk factor for serious secondary headache. However, the initial hypothesis that copeptin would rule out serious secondary headache at the predefined cutoff of 5.0 pmol/L and rule in serious secondary headache at 20 pmol/L with a high sensitivity and specificity could “as is” not be confirmed. But at a lower cutoff level of 2.5 pmol/L, copeptin had a sensitivity of > 90%.

The most commonly postulated mechanism for the early copeptin elevation in acute illness is that copeptin reflects the activation of the hypothalamus-pituitary-adrenal axis at the hypothalamic level [41]. Copeptin promptly rises in different physical stress situations, correlating with the magnitude of stress [41–43]. Consequently, copeptin has been shown to be useful for an early rule out of acute myocardial infarction [29] and for prognosis in acute ischemic stroke [31]. We now show that copeptin may also be a promising marker for ruling out serious secondary headaches.

In acute headache with its high burden of disease [44], no biomarker has been established in clinical routine to improve discrimination between serious secondary and benign headache forms, even though fast and early identification of serious secondary headaches at the ED is crucial and has remained an unmet clinical need [2]. Algorithms based on clinical features do exist, but are inconsistently applied; [2] thus, there is room for improvement. Some biomarkers have been explored when specific causes of secondary headache were suspected, such as D-dimers for ruling-out sinus vein thrombosis [14]. Furthermore, there have been efforts for biomarker-guided treatment in migraine, for example by predicting treatment response to triptans by measuring levels of calcitonin gene-related peptide or neurokinin A [22]. Based on our data, copeptin seems to have potential as a biomarker in unselected patients presenting with acute non-traumatic headache to the emergency department.

However, as copeptin rises after stress independent from its cause, potential confounding factors have to be ruled out, especially extra-neurologic causes such as systemic inflammation. Further studies seem warranted which analyze the influence of stress and pain itself on copeptin values before copeptin may be introduced into clinical decision-making.

Interestingly, male gender was independently associated with serious secondary headache in multivariate analysis. We attribute this finding to the fact that primary headache, especially migraine, is more frequent in women [45].

Even though copeptin has been shown to predict adverse outcome in various diseases [46, 47], there was only a trend for the association with the combined endpoint of death or hospitalization in this study after adjusting for all other relevant risk factors. Copeptin was not associated with mortality or hospitalization in multivariate analysis. When looking only at mortality, copeptin levels were higher in nonsurvivors as compared to survivors. However, due to the very small number of nonsurvivors (*n* = 2), this finding needs further evaluation in larger cohorts.

Copeptin seems therefore not to be an ideal marker to identify patients in need for hospitalization, probably because there are other factors contributing to hospitalization which are not sufficiently reflected by copeptin levels, such as psychosocial factors (e.g., need for nursing care, patients’ fears of worsening or uncontrollable pain), comorbidities, or organizational reasons (observation until further investigations were performed, i.e. at night and on week-ends).

The greatest potential of copeptin in the studied setting is to rule out serious headache forms by its high negative predictive value with a similar accuracy as the clinical rule based on “red flags”. We speculate that the reason lies in the non-specific and prompt elevation of copeptin in stress situations. Besides the underlying disease in secondary headache forms, pain itself as a stress stimulus may have contributed to the increase in copeptin levels. This would explain why some individual patients with migraine also had very high copeptin values. So far, there is only data on copeptin levels in relation to chest pain but not to other pain stimuli. In chest pain, copeptin levels are higher in acute myocardial infarction than in extracardiac chest pain [29]. Thus, if pain stimuli are rather small and other relevant stress stimuli are lacking, low copeptin levels should accurately identify benign headache forms.

## Conclusion

Copeptin was independently associated with serious secondary headache as compared to benign headaches forms. Copeptin may be a promising novel blood biomarker for risk stratification in patients with non-traumatic headache.

## Appendix

### Definition of serious secondary NTH

A serious NTH will require subsequent treatment of the underlying disease or condition. If untreated, the patient faces the risk of permanent damage or death.

The following diagnoses were selected from the list of secondary NTH in the ICHD-II criteria [9] as meeting the above criterion:

- [G44.81] Headache attributed to cranial or cervical vascular disorder, i.e. the following:

Headache attributed to

- ischaemic stroke or transient ischaemic attack
- non-traumatic intracranial haemorrhage
- unruptured vascular malformation
- vasculitis
- cerebral venous thrombosis (CVT)
- other intracranial vascular disorder
- Headache or facial or neck pain attributed to arterial dissection

- [G44.813] Headache attributed to arterial hypertension, i.e. the following:

Headache attributed to

- phaeochromocytoma
- hypertensive crisis without hypertensive encephalopathy
- hypertensive encephalopathy
- pre-eclampsia
- eclampsia
- other

- [G44.82] Headache attributed to non-vascular intracranial disorder, i.e. the following:

- high cerebrospinal fluid pressure
- idiopathic intracranial hypertension (IIH)
- intracranial hypertension secondary to metabolic, toxic or hormonal causes
- intracranial hypertension secondary to hydrocephalus
- low cerebrospinal fluid pressure
- epileptic seizure
- other non-vascular intracranial disorder

- [G44.82] Headache attributed to non-infectious inflammatory disease, i.e. the following:

Headache attributed to

- neurosarcoidosis
- aseptic (non-infectious) meningitis
- lymphocytic hypophysitis
- other non-infectious inflammatory disease

- [G44.822] Headache attributed to intracranial neoplasm
- [G44.4 or G44.83] Headache attributed to a substance or its withdrawal, i.e. the following:

- Carbon monoxide-induced headache
- Cocaine-induced headache
- Other

- [G44.821] Headache attributed to intracranial infection
- [G44.84] Headache or facial pain attributed to disorder of cranium, neck, eyes, ears, nose, sinuses, teeth, mouth or other facial or cranial structures, i.e. the following:

- Headache attributed to acute glaucoma
- Other

- [G44.847, G44.848 or G44.85] Cranial neuralgias and central causes of facial pain, i.e. the following:

- Optic neuritis
- Head or facial pain attributed to acute herpes zoster
- other

## Additional file

Additional file 1: Spreadsheet of study data. (XLSX 57 kb)

## Abbreviations

AUC: Area under the curve
CI: Confidence interval
ED: Emergency Department
ICHD: International Classification of Headache Disorders
IQR: Interquartile range
ROC: Receiver operating characteristic
VAS: Visual analogue scale
